# Utility of global longitudinal strain in early identification of chronic cardiotoxicity in asymptomatic long-term malignant lymphoma survivors with normal left ventricle ejection fraction

**DOI:** 10.1038/s41598-025-93933-2

**Published:** 2025-03-18

**Authors:** Eva Rihackova, Michal Rihacek, Lubos Boucek, Maria Vyskocilova, Lubomir Elbl

**Affiliations:** 1 Department of Internal Medicine and Cardiology, University Hospital Brno and Faculty of Medicine, Masaryk University, Brno, Czech Republic; 2 Department of Pharmacology, Faculty of Medicine, Masaryk University, Brno, Czech Republic; 3 Department of Laboratory Methods, Faculty of Medicine, Masaryk University, Brno, Czech Republic; 4 Department of Hematology and Transfusion, AGEL Hospital Prostejov, Prostejov, 796 01 Czech Republic

**Keywords:** Cardiotoxicity, Global longitudinal strain, Anthracyclines, Radiotherapy, Echocardiography, Cardiology, Medical research

## Abstract

Malignant lymphoma survivors are at increased risk for anthracycline and/or radiotherapy-induced chronic cardiotoxicity. Proper long-term follow-up is essential for malignant lymphoma survivors after-care. This study aimed to assess TTE parameters of potential subclinical cardiotoxicity and to examine their utility in diagnosing chronic cardiotoxicity. Improvement of the diagnostic process may precede the manifestation of cardiac adverse events. Main objective of the study was to improve the identification of cancer survivors in increased risk of treatment cardiotoxicity. To achieve this goal, utility of various echocardiography parameters was examined.In this retrospective study we analysed TTE of 167 subjects with speckle tracking according to the European Society of Echocardiography guidelines during the follow-up period. 88 of them were long-term lymphoma survivors diagnosed with malignant lymphoma between the years 1994–2015. Minimum follow up period was 5 years with the median of 10 years after anti-cancer treatment cessation. TTE were performed between the years 2017–2022 at cardio-oncology outpatient office during regular follow-up period. A total of 79 volunteers with no history of chronic heart failure (CHF) or decline in LVEF, 51 (64.6%) of whom were males, with the median age of 46 (16–58) years were included in the analysis as control group. Control subjects had various indications for TTE (e.g. preoperative examination, benign palpitations, or with well controlled arterial hypertension taking two antihypertensives at most). Ischemic heart disease was ruled out by stress test. None of the control subjects had history of stroke or chronic lower limb ischemia. All control subjects were considered clinically stable with no sign of cardiac impairment caused by primary disease. Both cancer survivors and control group were divided into subgroups based on LVEF: lower normal LVEF (53–61%), and higher normal LVEF (> 61%). Survivors with lower normal LVEF (53–61%) had a statistically significant decline in GLS compared to those with higher normal LVEF (> 61%). This phenomenon was not observed in control group indicating a possible additional diagnostic value of this parameter. Inclusion of GLS assessment in follow-up TTE examination of subjects with lower normal LVEF may improve the sensitivity of detection of chronic cardiotoxicity. Patients with declined GLS and lower normal LVEF are candidates for intensified follow-up to precede manifestation of cardiac adverse events.

## Introduction

Both modern and old anti-cancer therapeutic modalities (especially anthracyclines and thoracic irradiation) used in the treatment of malignant lymphomas possess cardiotoxic potential. Although cardiotoxicity of a treatment of malignant lymphoma can be both acute and chronic, where chronic is represented by two main categories early-onset (type I) and late-onset (type II), it is the Type I, which occurs within one year after chemotherapy cessation. Type II cardiotoxicity may manifest after the first year with an unlimited timeframe^1^. Cardiotoxicity is defined as a drop in left ventricular ejection fraction (LVEF) by 10%. The absolute lower limit for LVEF differs, some authors point out the value < 50%, according to expert consensus of the American Society of Echocardiography and the European Association of Cardiovascular Imaging < 53%^2,3^.

The initiation of cardioprotective therapy based on LVEF alone has limitations due to its low sensitivity. On the other hand, global longitudinal strain (GLS) is a robust and sensitive marker of left ventricular impairment^4^. Evaluation of GLS may prove useful in early detection of cardiac toxicity caused by oncology treatment^5^. Whether GLS may serve as “a robust” tool in long-term follow-up of type II cardiotoxicity is yet to be determined.

Chronic cardiotoxicity represents an immense problem considering the increasing number of survivors due to the progress in anti-cancer treatment. This is particularly seen in young-age cancer survivors, e.g. Hodgkin lymphoma (HL)^6,7^.

**Fig. 1:**
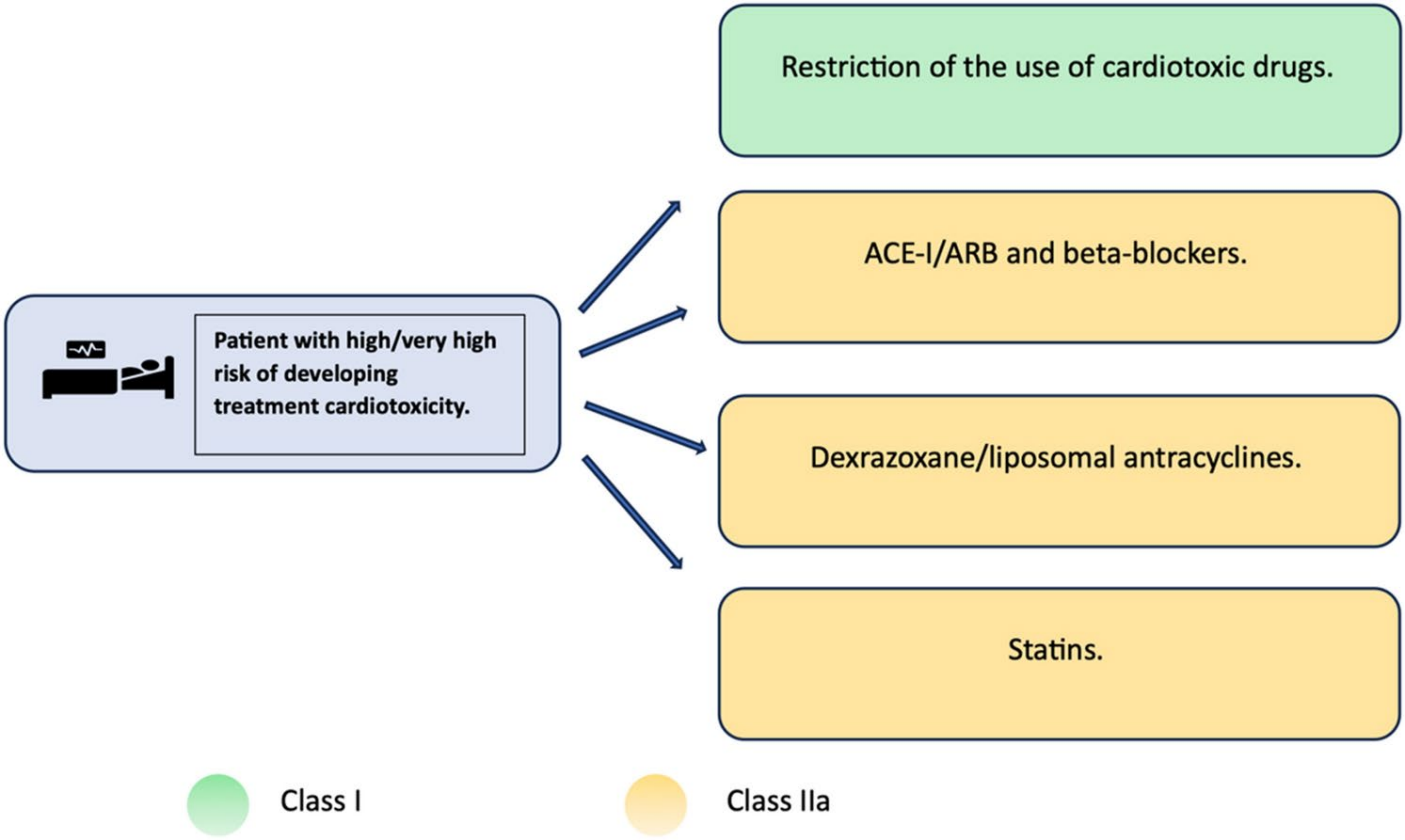
General strategy and recommendations for reducing the risk of developing cardiotoxicity in patients at high risk of cardiotoxicity associated with antitumor therapy. ACE-I – angiotensin-converting enzyme inhibitors; ARB – AT1 receptor blockers for angiotensin II; ESC – European Society of Cardiology; CVD – cardiovascular disease; RF – risk factor.

Primary prevention is represented by modification of drug formula (e.g liposomal form of anthracyclines, modern irradiation techniques), or using an alternative chemotherapeutics with similar efficacy and lower cardiac burden. Another option is administration of cardioprotective agents^1,8^. In terms of primary cardioprotection for patients at high or very high risk of cardiotoxicity, the administration of statins should be considered. For patients in this group who are additionally treated with targeted therapy (that is known to possibly cause heart failure) or anthracyclines, therapy with beta-blockers in combination with ACE inhibitors or ARBs should be considered. Furthermore, for patients in this group treated with high-dose anthracyclines, the administration of dexrazoxane should be considered (see Fig. 1). The mentioned recommendations have a low level of endorsement, and there are currently no entirely convincing data on the benefits of this treatment approach^9^. However, several clinical trials on cardioprotection during anti-cancer treatment are currently conducted including STOP-CA with atorvastatin/DOX. Early results of STOP-CA presented by Neilan TG at ACC/WCC 2023 show that patients receiving 40 mg of oral atorvastatin were less likely to develop 10% or greater decline in LVEF than those receiving placebo^10^. Clinical trial evaluating the effects of ACE-i and β-blockers in management of cardiotoxicity in cancer patients are expected to be completed in 2030^11^. Until chemotherapeutics with cardiac adverse effects are incorporated into clinical practice, comprehensive long-term follow-up, prompt initiation of farmacotherapy for heart failure will be essential for inpatient care^9^.

According to ESC Cardio-oncology guidelines, survivors are divided into two groups of risk categories according to maturity during exposition to potentially cardiotoxic agents: 1/ childhood and adolescent cancer survivors (CACS) and 2/ adult cancer survivors (AdCS). Furthermore, the subjects are divided into subgroups based on chosen therapeutic regimens, baseline cardiovascular toxicity risk, or whether they experienced cancer-therapy-related cardiac dysfunction during treatment^9^. Most childhood adolescent-age survivors are classified as high-risk or very high-risk, where echocardiography is recommended every two years^9,12^.

**Fig. 2:**
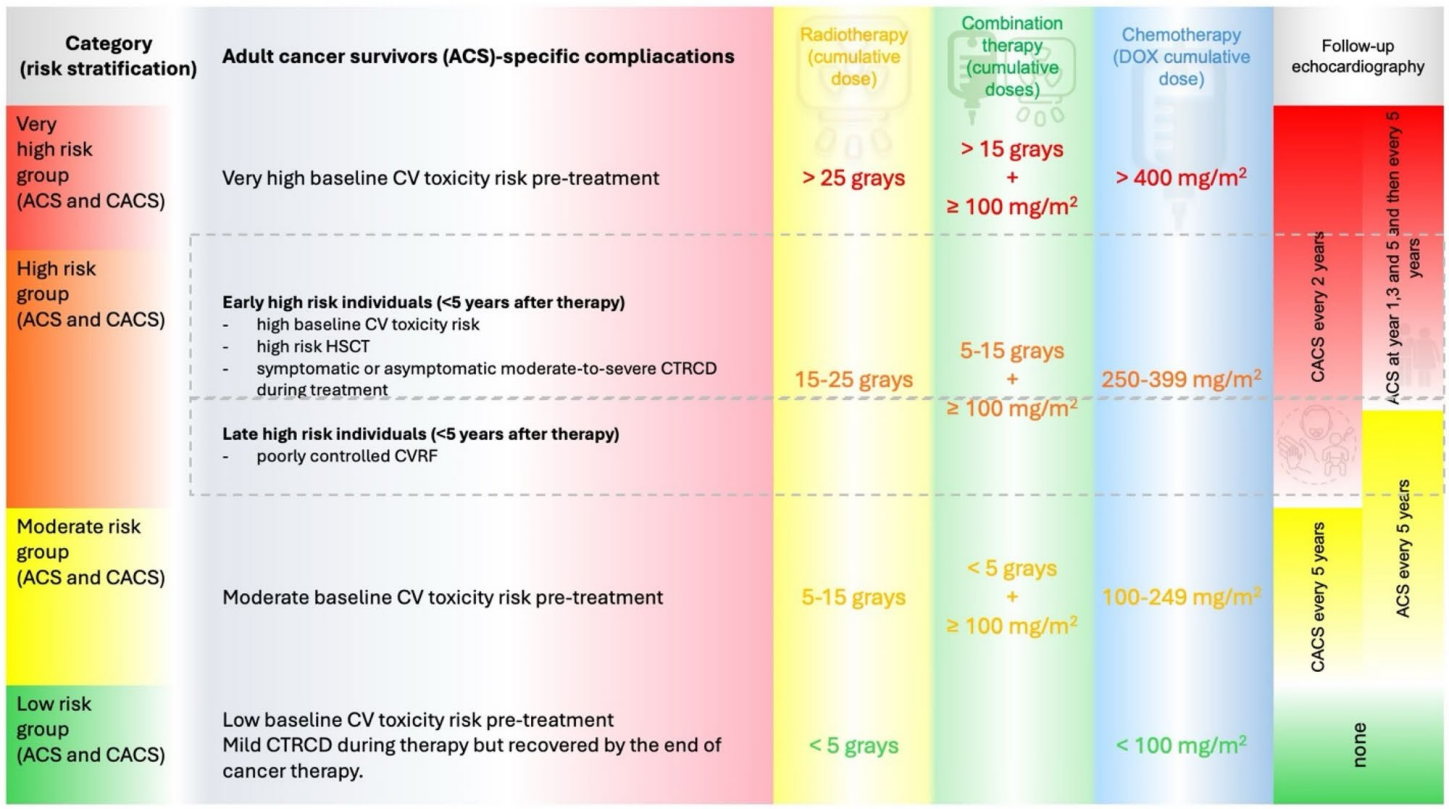
Follow-up TTE recommendations according to ESC Cardio-oncology guidelines 2022. Abbreviation: adult cancer survivors (AdCS), childhood and adolescent cancer survivors (CACS).

The median age of patients at the diagnosis of non-Hodgkin lymphoma (NHL) is 67 years, whereas HL is commonly diagnosed between 20 and 34 years of age^13^. Most lymphoma patients are classified as AdCS receiving higher doses of anthracyclines or radiotherapy and are therefore indicated to have an echocardiographic screening at years 1,3 and 5 after the cessation of anti-cancer therapy with subsequent examinations every 5 years. However, only a weak recommendation for echocardiographic screening exists in moderate-risk AdCS and late high-risk AdCS^9^. Follow-up recommendations based on 2022 ESC cardio-oncology guidelines are described in more detail in Fig. 2.

Echocardiography screening for cardiac impairment in HL survivors is recommended every 5 years (in lower-risk patients, guidelines even allow omission of echocardiography screening). This period represents a long interval in which subclinical cardiac impairment may progress into symptomatic heart failure. In practice, routine follow-up after anti-cancer treatment performed in all cases may provide us with information about cardiac function. However, in terms of sensitive identification of the most vulnerable long-term survivors, basic TTE parameters such as LVEF may prove insufficient. Therefore, parameters evaluating slight signs of cardiac impairment, such as GLS could prove useful in the identification of these cases. In terms of GLS evaluation, performing a full examination on every subject would be time-consuming hence subjects would have to be chosen carefully according to previously selected criteria.

This study aimed to assess LVEF in correspondence with GLS obtained from echocardiographic findings in clinically asymptomatic long-term malignant lymphoma survivors. We focused on the evaluation of GLS in patients with normal LVEF to examine its possible benefit in more sensitive identification of early cardiac impairment and subclinical cardiotoxicity.

## Materials and methods

### Study design

In this retrospective single tertiary care centre study, medical charts were reviewed from the cardio-oncology outpatient office at the Department of Internal Medicine and Cardiology, Faculty Hospital Brno between the years 2017 and 2022. All experimental protocols were approved by the local ethical board of the University Hospital Brno 03–100,424/EK. All methods were carried out in accordance with relevant guidelines and regulations. Informed consent was waived by the local ethical board of the University Hospital Brno 03–100,424/EK.

### Transthoracic echocardiography (TTE)

TTE was performed using the GE Vivid E90 or E95 systems, following the guidelines of the European Society of Echocardiography^14^. We calculated LVEF using Simpson’s biplane method. TTE was performed by a single experienced professional with long-term practice in echocardiography and clinical cardiology. Global longitudinal strain (GLS) was assessed by speckle tracking in apical long-axis view (GLS_APLAX), four-chamber view (GLS_4CH), 2-chamber view (GLS_2CH), and by an average of GLS values obtained from all three planes (GLS_AVG). Besides standard TTE parameters, the myocardial performance index (MPI) was assessed. The MPI is calculated as a sum of isovolumetric contraction time (ICT) and isovolumetric relaxation time (IRT) divided by ejection time. Value of LVEF of < 53 was considered abnormal^2,3^. The cutoff value for normal left ventricular ejection fraction (LVEF) of 53% was selected based on the recommendations of Plana et al. to address concerns that the ESC-recommended cutoff of 50% (50–53% respectively, which had previously been considered borderline) may include patients with potentially affected GLS^15^.

### Characteristics of the studied group

A total of 167 subjects attending the cardiology outpatient cardio-oncology office at University Hospital, Brno, Czech Republic for routine check-ups with TTE examinations were included in the study.

### Characteristics of the long-term survivors group

A total of 88 adult lymphoma survivors with the median age of 40 (24–84) years were enrolled in the analysis. They were diagnosed with malignant lymphoma between the years 1994–2015. In this group, 46 (52.3%) patients were males and 42 (47,7%) were females. The median age at primary oncological diagnosis was 29 (15–76). A cardiovascular examination was carried out 10 (5–27) years after treatment. The underlying diagnosis was Hodgkin lymphoma in 79 (89.8%) cases and non-Hodgkin lymphoma in 9 (10.2%).

All of the survivors were treated with DOX, the median cumulative doxorubicin (DOX) dose applied during treatment was 200 (50–400) mg/m^2^. While combination therapy (DOX + ionizing radiation) received 53 (60.2%) cases with the median cumulative dose of 30 (20–50)Gray.

### Characteristics of the control group

The group consisted of subjects with various indications for TTE (e.g. preoperative examination, benign palpitations, or with well controlled arterial hypertension taking two antihypertensives at most). Ischemic heart disease was ruled out with stress test, they did not have history of stroke or chronic lower limb ischemia. When enrolled, all subjects were considered clinically stable with no sign of cardiac impairment caused by primary disease. A total of 79 volunteers with no history of chronic heart failure (CHF) or decline in LVEF, 51 (64.6%) of whom were males, with the median age of 46 (16–58) years were included in the analysis.

General characteristics of the studied group and the control group are summarized in Table 1.

**Table 1:** General characteristics of the studied group and the control group including cardiovascular risk factors and data about ambulatory cardiovascular medication.

**Characteristic**	**control** N = 79^*1*^	**survivor** N = 88^*1*^
HT	27 (34%)	12 (13.6%)
ICHS	0 (0%)	3 (3.4%)
HLP	19 (24%)	5 (5.7%)
DM	5 (6.3%)	4 (4,5%)
BB	0 (0%)	5 (5.7%)
ASA	0 (0%)	2 (2.3%)
OAC/DOAC	13 (16%)	1 (1.1%)
ACEI/ARB	16 (20%)	11 (12.5%)
CCB	4 (5.1%)	4 (4.5%)
DIU	2 (2.5%)	5 (5.7%)
INZ	2 (2.5%)	1 (1.1%)
OAD	3 (3.8%)	3 (3.4%)
STATINS	16 (21,5%)	5 (5,7%)
^*1*^n (%)		

HT: Hypertension ICHS: Ischemic Heart Disease HLP: Hyperlipidemia DM: Diabetes Mellitus BB: Beta-Blockers ASA: Acetylsalicylic Acid (Aspirin) OAC/DOAC: Oral Anticoagulants/Direct Oral Anticoagulants ACEI/ARB: Angiotensin-Converting Enzyme Inhibitors/Angiotensin II Receptor Blockers CCB: Calcium channel blockers DIU: Diuretics INZ: Insulin OAD: Oral antidiabetics.

### Statistical analysis

Variables with normal distribution were described by mean and standard deviation, otherwise, median and range were used.

The Shapiro–Wilk test was used to assess the normality of distribution. The independent t-test and the Mann–Whitney U test were used to compare groups with normal and non-normal distribution, respectively.

The Fisher’s exact test was used to compare proportions of pathological values.

The analyses were performed using the statistical software R (R Core Team, 2022).

## Results

Survivors had statistically significant lower LVEF and significantly impaired GLS_AVG and GLS_APLAX comparing to control group, see Table 2.

**Table 2:** Comparison of survivors and control group parameters. Abbreviations: age at the time of diagnosis (AGE_DG), body mass index (BMI), Gray (Gy), Left ventricular mass index (LVMI), myocardial performance index (MPI), tricuspid annular plane systolic excursion (TAPSE).

Survivors and control groups			
Parameter	Survivors	Control	p-value
Age (years)	40 (24–84)	46 (16–58)	0.12
Age_DG (years)	29 (15–76)	–	–
BMI (kg/m^2^)	22.8 (16.7–44.3)	26.4 ± 4.2	< 0.001
DOX (mg/m^2^)	200 (50–400)	–	–
LVEF (%)	65.0 (36.0–74.0)	67.9 ± 4.1	< 0.001
Follow-up (years)	10 (5–27)	NA	
GLS_2CH (%)	−19.2 ± 3.4	−20 (−25 – −15)	0.17
GLS_4CH (%)	−19 (−27—−10)	−20 (−25—−14)	0.092
GLS_APLAX (%)	−18.9 ± 3.9	−20.2 ± 2.5	0.0099
GLS_AVG (%)	−19 (−27—−10)	−20 (−24—−16)	0.017
Irradiation (Gy)	30 (0–50)	–	–
LVMI (g/m^2^)	89.5 (43–152)	90.9 ± 18.8	0.51
MPI (ratio)	0.60 ± 0.11	0.62 ± 0.11	0.26
TAPSE (mm)	23 (15–35)	23 (18–32)	0.57

**Fig. 3:**
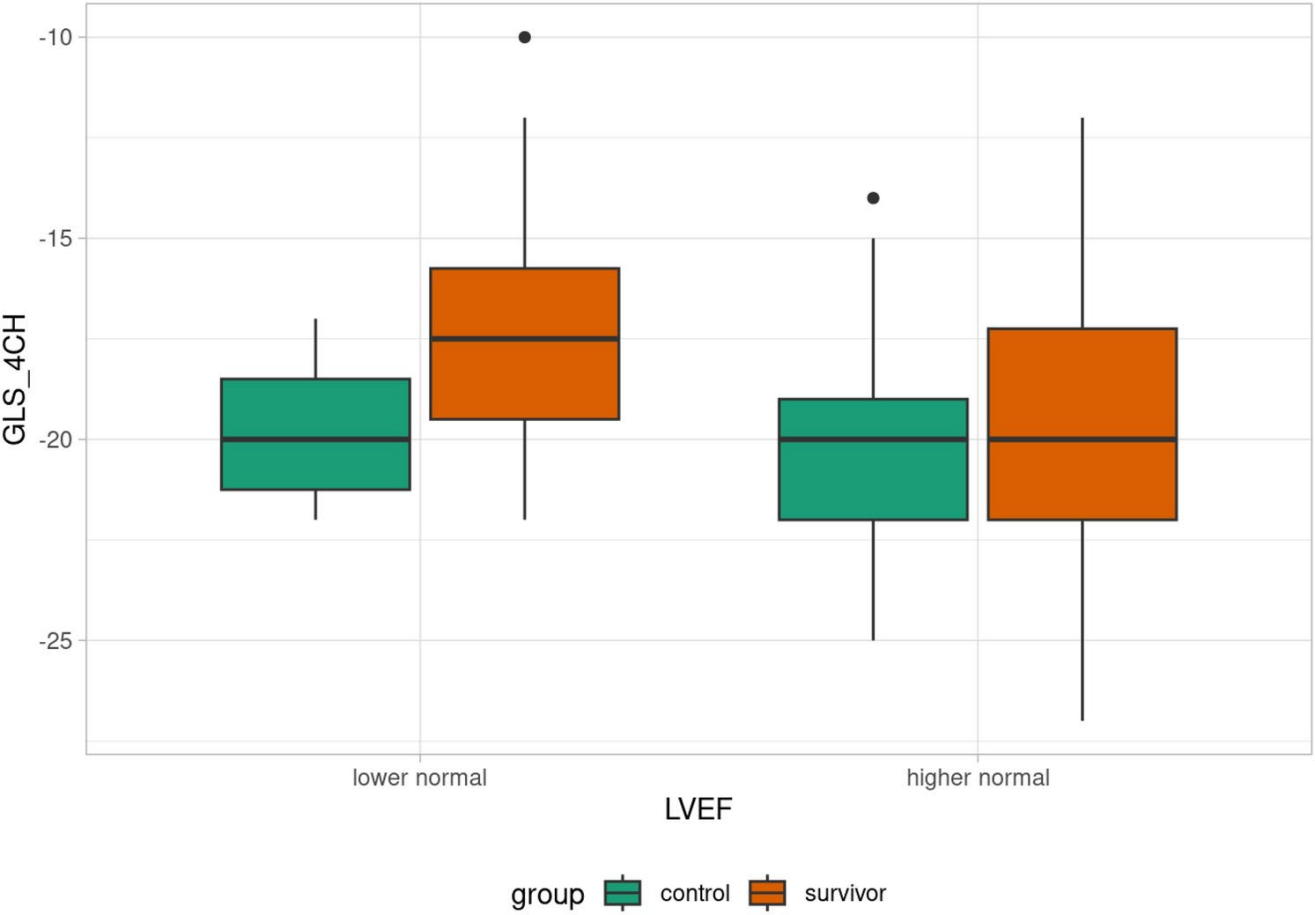
Box plot of GLS_4CH value according to LVEF in control/survivor group.

**Fig. 4:**
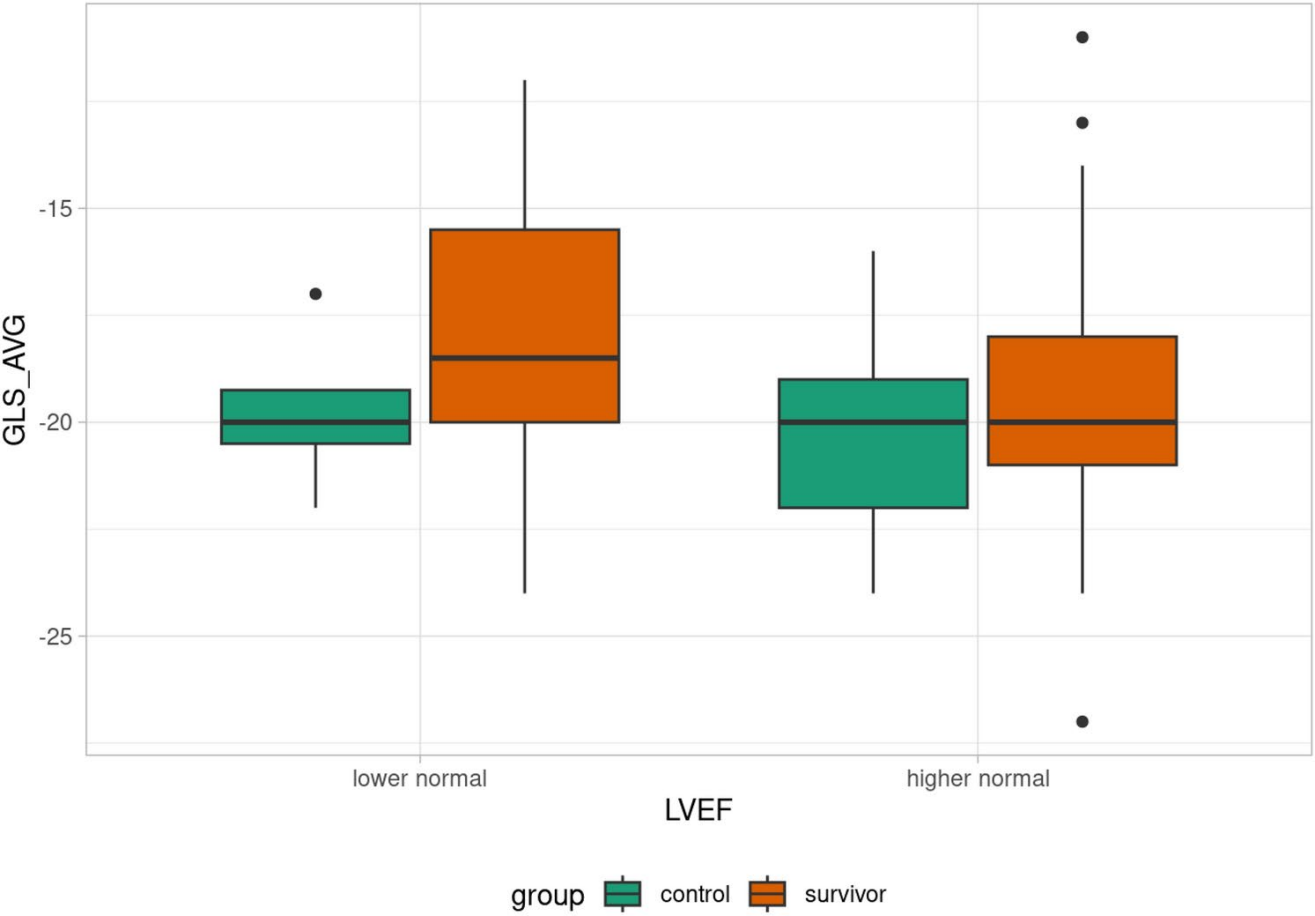
Box plot of GLS_AVG value according to LVEF in control/survivor group.

**Fig. 5:**
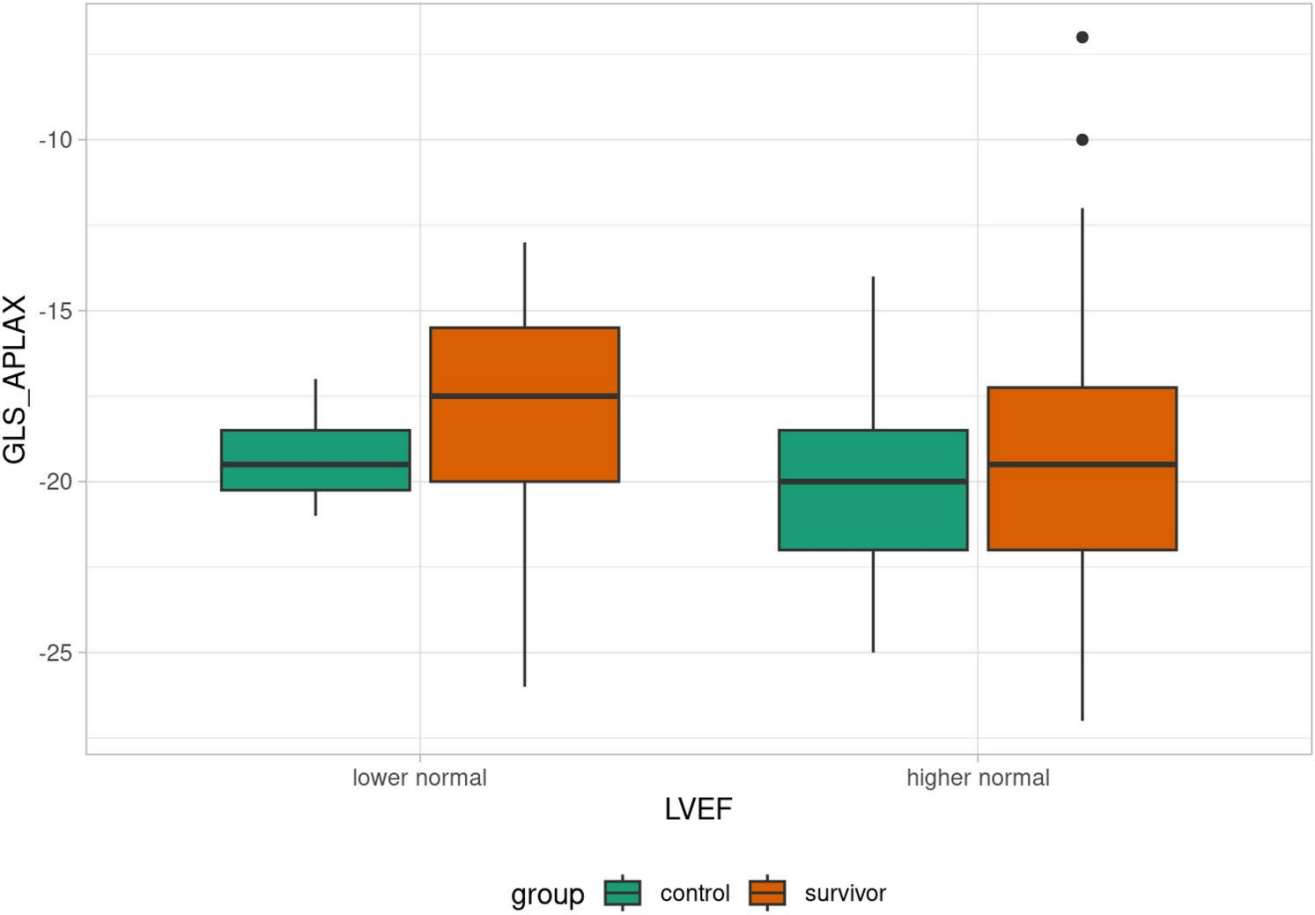
Box plot of GLS_APLAX value according to LVEF in control/survivor group.

**Fig. 6:**
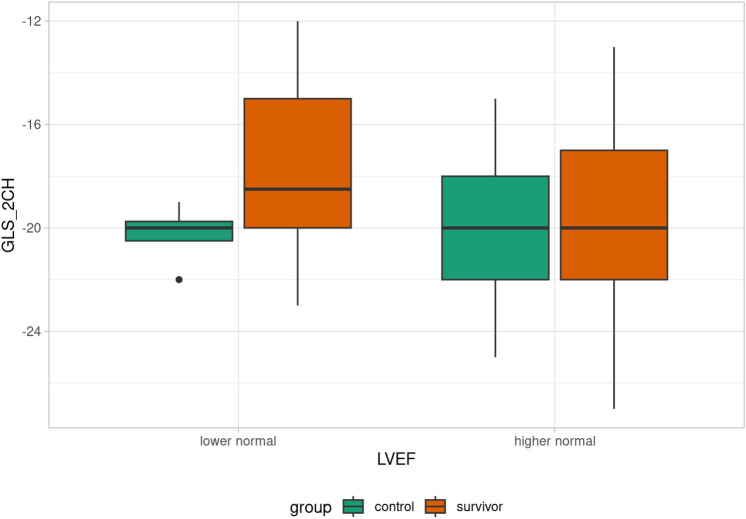
Box plot of GLS_2CH value according to LVEF in control/survivor group.

Patients were divided into subgroups based on LVEF: decreased LVEF (< 53%), lower normal LVEF (53–61%), and higher normal LVEF (> 61%). One survivor had decreased LVEF, 17 survivors had lower normal LVEF, and the rest of the survivors had higher normal LVEF. Differences in GLS in each plain between the lower normal LVEF and higher normal LVEF groups were evaluated. Statistically significant differences between the lower normal and higher normal subgroups were found in GLS parameters GLS _2CH (p = 0.022), GLS _4CH (p = 0.023), GLS _AVG (p = 0.043), see Table 3. That difference was not observed in the control group (Table 4). Comparison of GLS value in each plain according to LVEF in control/survivor group is summarized in Figs 3,4,5,6.

**Table 3:** GLS evaluation in lower normal LVEF, and higher normal LVEF subgroups (survivor group). Abbreviations: GLS (global longitudinal strain), LVEF (left ventricle ejection fraction), GLS_APLAX (global longitudinal strain apical long-axis view), GLS_4CH (global longitudinal strain four-chamber view), GLS_2CH (global longitudinal strain 2-chamber view), GLS_AVG (an average of GLS values obtained from all three planes).

GLS evaluation in lower normal LVEF, and higher normal LVEF subgroups (survivor group)			
	**Lower normal LVEF**	**Higher normal LVEF**	**p-value**
GLS_2CH (%)	−17.6 ± 3.1	−19.7 ± 3.2	0.022
GLS_4CH (%)	−17.2 ± 3.5	−20 (12–27)	0.023
GLS_APLAX (%)	−17.8 ± 3.5	−19.3 ± 3.8	0.14
GLS_AVG (%)	−17.6 ± 3.2	−19.4 ± 2.9	0.043

**Table 4:** GLS evaluation in lower normal LVEF, and higher normal LVEF subgroups (control group). Abbreviations: GLS (global longitudinal strain), LVEF (left ventricle ejection fraction), GLS_APLAX (global longitudinal strain apical long-axis view), GLS_4CH (global longitudinal strain four-chamber view), GLS_2CH (global longitudinal strain 2-chamber view), GLS_AVG (an average of GLS values obtained from all three planes).

GLS evaluation in lower normal LVEF, and higher normal LVEF subgroups (control group)			
	**Lower normal LVEF**	**Higher normal LVEF**	**p-value**
GLS _2CH (%)	−20.3 ± 1.3	−20 (15–25)	0.78
GLS _4CH (%)	−19.8 ± 2.2	−20 (14–25)	0.88
GLS _APLAX (%)	−19.3 ± 1.7	−20.2 ± 2.5	0.34
GLS _AVG (%)	−19.8 ± 2.1	−20 (16–24)	0.69

Comparing patients treated with combined therapy of DOX and ionizing irradiation, and those treated solely with DOX, we found statistically significant differences in LVMI (p = 0.0013), MPI (p = 0.01), TAPSE (p = 0.037), see Table 5.

**Table 5:** Comparison of survivors and control group parameters. Abbreviations: age at the time of diagnosis (AGE_DG), body mass index (BMI), Gray (Gy), Left ventricular mass index (LVMI), myocardial performance index (MPI), tricuspid annular plane systolic excursion (TAPSE).

Irradiated, and unirradiated subgroups			
Parameter	**Unirradiated**	**Irradiated**	**p-value**
Age (years)	46.7 ± 14.5	37.0 (24–68)	0.014
Age_DG (years)	37 (17–76)	22.5 (16.9–35.8)	0.0015
BMI (kg/m^2^)	23.1(16.7–44.3)	26.4 ± 4.2	0.049
DOX (mg/m^2^)	210 (50–400)	200 (70–400)	< 0.001
LVEF (%)	65.8 ± 3.8	64 (36–73)	0.15
Follow-up (years)	8 (5–27)	11 (5–24)	< 0.001
GLS_2CH (%)	−19.3 ± 3.2	−19.2 ± 3.6	0.87
GLS_4CH (%)	−19.2 ± 3.1	−19 (−27- −11)	0.75
GLS_APLAX (%)	−19.3 ± 3.5	−18.6 ± 4.1	0.42
GLS_AVG (%)	−19.3 ± 2.9	−19 (10–27)	0.7
Irradiation (Gy)	–	30 (20–50)	–
LVMI (g/m^2^)	99.0 ± 23.4	82.5 ± 21.4	0.0013
MPI (ratio)	0.6 ± 0.1	0.6 ± 0.1	0.01
TAPSE (mm)	24.9 ± 4.7	22.8 ± 4.2	0.037

## Discussion

Cardiotoxicity of anti-cancer therapy has been a topic since the 70 s when long-term adverse events of chemotherapy administered to individuals in the late 40 s and 50 s emerged and could be evaluated^16^. Since this initial period, advances and modernization of anti-cancer therapy led to a significant increase in long-term cancer survivors that require follow-up^17^. ESC 2022 Cardio-oncology guidelines present an overview and recommendations regarding risk stratification and long-term follow-up^9^, however, the topic of cardioprotective treatment adjustment is still steadily being discussed^1,18,19^.

### Global longitudinal strain in long-term cancer survivors

Assessment of GLS proved to be a valuable diagnostic parameter in the prediction of subclinical cardiac dysfunction and the prediction of a future significant LVEF decrease in patients with acute cardiotoxicity^20^. Nevertheless, few data regarding GLS use in the prediction of long-term cardiotoxicity and decline of LVEF in cancer survivors were published^21–23^.

Our study reports the results of a TTE examination in a group of clinically asymptomatic cancer survivors (median age at the diagnosis 29 years) in long-term follow-up (median 10 years). All subjects were classified as NYHA functional class I. Long-term CS had significantly lower LVEF, GLS_APLAX, and GLS_AVG compared to the control group. The significance of this finding is also supported by the higher average age of the control group compared to the long-term CS group at the time of TTE.

**Fig. 7:**
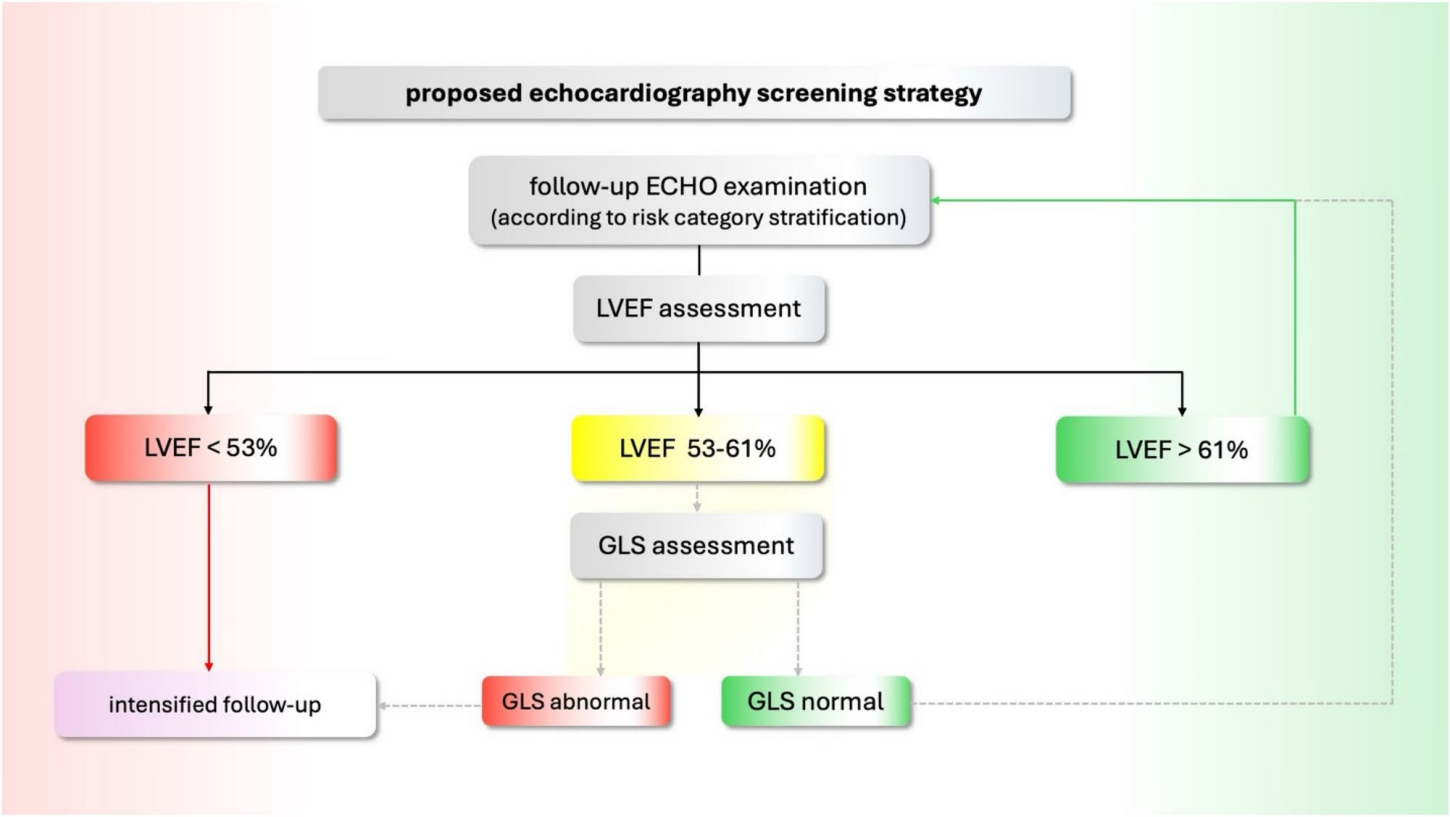
Proposed inclusion of GLS in the management of long-term CS TTE follow-up protocol.

In GLS analysis between the patients with lower normal LVEF (53–61%), and higher normal LVEF (> 61%), a significant difference was discovered in parameters: GLS _2CH, GLS _4CH, GLS _AVG. Decline in GLS was not found in the control group. This establishes a lower normal value of LVEF as a possible “red flag” and an indication for GLS evaluation in long-term cancer survivors. Considering our results and those previously published in the literature, we propose the inclusion of GLS in TTE follow-up protocol for long-term CS (depicted in Fig. 6) (Fig. 7).

To date, we have not found a study involving long-term AdCS classified as NYHA functional class I and no history of cardiac disease to compare with. A study involving AdCS with a history of breast cancer treatment (anthracycline and radiotherapy) in GLS evaluation was published but that study included around 50% of subjects with clinical signs of CVD^24^.

Our results are predominantly supported by studies involving CACS. A study by Gonzalez-Manzanares et al. identified a significantly higher number of acute lymphoblastic leukemia (ALL) CACS (median age at the diagnosis 4 years) suffering from subclinical cardiotoxicity by GLS than by LVEF assessment^21^. Another study by Niemelä et al. demonstrated that CACS with various malignancies (age at the diagnosis mostly around 8 years), a history of anthracycline therapy, and abnormal LV longitudinal strain had normal LVEF in 70% of cases. This suggests a potential of GLS for long-term risk assessment among CACS beyond the first decade of follow-up^22^. Furthermore, GLS seemed to be a more sensitive parameter for determining LV dysfunction than LVEF in the study by Christiansen et al. on CACS (age at the diagnosis mostly around 5 years) with a history of childhood lymphoma and acute leukemia^23^.

No relation of LVEF and GLS to the DOX dose was discovered, the latter being consistent with the results of a study by Wolf et al.^12^. This phenomenon could be explained by possible individual susceptibility to DOX-induced cardiac damage or an insufficient number of subjects in our study. Furthermore, our evaluation is limited because the study lacked long-term CS with no history of DOX.

In 2017 a model examining relation between LVEF, and myocardial strain was published by Stokke et al. This study elucidated, why simultaneous normal LVEF and significantly altered LV function may coexist. According to this model LVEF is quadratically dependent on global circumferential strain (GCS) though linearly on GLS and even a significant decline in GLS may be compensated with GCS. Furthermore, Stokke’s model explains how LVEF may be affected by ventricle size and wall thickness. It was found, that reduced ventricle size with thicker wall amplifies each other’s impact on LVEF^25^. Thus, increasing myocardial wall thickness and simultaneous reduction of ventricle size results in stabile LVEF despites highly affected GCS.

. Considering, that radiotherapy-induced heart damage leads to decrease in LVMI (as also demonstrated in our study) and concentrical remodulation, GLS assessment, especially in cancer survivor’s cohort, may lead to sooner detection of cardiac impairment. Therefore, introduction of GLS into chronic cardiotoxicity follow-up protocols may prevent underdiagnosing post-radiotherapy systolic cardiac dysfunction^26,27^.

### Another assessed TTE parameters

Comparison between long-term cancer survivors with a history of radiotherapy + DOX and those with a history of DOX and no radiotherapy revealed significantly worse function of the right ventricle—TAPSE, lower LVMI, and worse MPI in subjects with a history of radiotherapy. The observed decline of LVMI is consistent with previously published studies and supports the theory of myocardial wall thinning caused by anti-cancer treatment-induced cardiomyocyte loss^13^. Although left ventricle impairment caused by anti-cancer therapy is well studied, the influence of irradiation on the function of the right ventricle is often overlooked. Anatomically, RV is exposed to high doses of irradiation. In our study, survivors who underwent irradiation had significantly worse TAPSE possibly caused by radiotherapy-induced RV remodelling^14^. The group treated with irradiation had significantly worse MPI. According to a recently published study from Dons et al. increasing MPI in patients with atrial fibrillation was associated with an increased risk of major cardiac adverse events^15^. Whether this parameter can be used as a predictor of major cardiac adverse events in patients without atrial fibrillation is yet to be evaluated.

On TTE, there were no abnormalities in morphologic parameter LVMI compared to controls supporting the evidence of subclinical rather than clinical cardiovascular impairment is consistent with other recently published studies^12^.

### Study limitations

One limitation of this study is its retrospective design, which may introduce selection bias and limit the ability to draw definitive causal inferences regarding the association between echocardiographic parameters and chronic cardiotoxicity in long-term malignant lymphoma survivors. Occurrence of malignant lymphoma in human population is heterogenous ranging from early life to the old age. Our study includes patients mostly from early adulthood with median of age 29 years. Age match between our survivor group and control group was not optimal, however GLS value in higher age (control group) is expected to be pathological more often than in adolescence which tightens up criteria for statistical significance. Other limitations are caused by survivor/selection bias, being a single centre study and limited number of samples.

## Conclusion

In this study, we discovered, that patients with lower normal LVEF (53–61%) had a statistically significant decline in GLS, indicating a possible diagnostic value of this parameter. Patients with declined GLS and lower normal LVEF are candidates for intensified follow-up to precede manifestation of cardiac adverse events. Further prospective randomised trials are necessary to confirm whether inclusion of GCS-led follow up will result in decreasing cardiovascular morbidity and mortality similarly to its utility in early detection of acute cardiotoxicity.

## Data Availability

The data presented in this study are available on request from the corresponding author.

## References

[1] Rihackova, E., Rihacek, M., Vyskocilova, M., Valik, D. & Elbl, L. Revisiting treatment-related cardiotoxicity in patients with malignant lymphoma-a review and prospects for the future. *Front. Cardiovasc. Med.* **10**, 1243531 (2023). 37711551 10.3389/fcvm.2023.1243531 PMC10499183

[2] Bloom, M. W. et al. Cancer therapy-related cardiac dysfunction and heart failure: Part 1: Definitions, pathophysiology, risk factors, and imaging. *Circ. Heart Fail.* **9**(1), e002661 (2016). 26747861 10.1161/CIRCHEARTFAILURE.115.002661 PMC4709035

[3] Plana, J. C. et al. Expert consensus for multimodality imaging evaluation of adult patients during and after cancer therapy: A report from the American Society of echocardiography and the European association of cardiovascular imaging. *J. Am. Soc. Echocardiogr.* **27**(9), 911–939 (2014). 25172399 10.1016/j.echo.2014.07.012

[4] Thavendiranathan, P. et al. Strain-guided management of potentially cardiotoxic cancer therapy. *J. Am. Coll Cardiol.* **77**(4), 392–401 (2021). 33220426 10.1016/j.jacc.2020.11.020

[5] Ye, L. et al. Myocardial strain imaging by echocardiography for the prediction of cardiotoxicity in chemotherapy-treated patients: A meta-analysis. *JACC Cardiovasc. Imaging.* **13**(3), 881–882 (2020). 31734206 10.1016/j.jcmg.2019.09.013

[6] Siegel, R. L., Miller, K. D., Fuchs, H. E. & Jemal, A. Cancer statistics, 2022. *CA Cancer J. Clin.* **72**(1), 7–33 (2022). 35020204 10.3322/caac.21708

[7] Erdmann, F. et al. Childhood cancer: Survival, treatment modalities, late effects and improvements over time. *Cancer Epidemiol.* **71**(Pt B), 101733 (2021). 32461035 10.1016/j.canep.2020.101733

[8] Lipshultz, S. E. et al. Cardiotoxicity and cardioprotection in childhood cancer. *Acta Haematol.* **132**(3–4), 391–399 (2014). 25228565 10.1159/000360238

[9] Lyon, A. R. et al. 2022 ESC guidelines on cardio-oncology developed in collaboration with the European Hematology Association (EHA), the European Society for Therapeutic Radiology and Oncology (ESTRO) and the International Cardio-Oncology Society (IC-OS). *Eur. Heart J.* **43**(41), 4229–4361 (2022). 36017568 10.1093/eurheartj/ehac244

[10] ClinicalTrials.gov. Bethesda (MD): National Library of Medicine (US). 2000 Feb 29 -. Identifier NCT02943590, STOP-CA (Statins TO Prevent the Cardiotoxicity From Anthracyclines) (2022). Available at: https://clinicaltrials.gov/ct2/show/study/NCT02943590?term=NCT02943590&draw=2&rank=1 (Cited 2023 May 8).as

[11] ClinicalTrials.gov. Bethesda (MD): National Library of Medicine (US). 2000 Feb 29 -. Identifier NCT02818517, Evaluation and Management of Cardio Toxicity in Oncologic Patients. (2022). Available at: https://clinicaltrials.gov/ct2/show/NCT02818517?term=NCT02818517&draw=2&rank=1 (Cited 2023 May 8).

[12] Wolf, C. M. et al. Subclinical cardiac dysfunction in childhood cancer survivors on 10-years follow-up correlates with cumulative anthracycline dose and is best detected by cardiopulmonary exercise testing, circulating serum biomarker, speckle tracking echocardiography, and tissue doppler imaging. *Front. Pediatr.* **8**, 123 (2020). 32296665 10.3389/fped.2020.00123 PMC7136405

[13] Lewis, W. D., Lilly, S. & Jones, K. L. Lymphoma: Diagnosis and treatment. *Am. Fam. Physician.* **101**(1), 34–41 (2020). 31894937

[14] Evangelista, A. et al. European association of echocardiography recommendations for standardization of performance, digital storage and reporting of echocardiographic studies. *Eur. J. Echocardiogr.* **9**(4), 438–448 (2008). 18579482 10.1093/ejechocard/jen174

[15] CSP L, SD S. Classification of Heart Failure According to Ejection Fraction: JACC Review Topic of the Week - PubMed. Journal of the American College of Cardiology. 06/29/2021 77(25) 10.1016/j.jacc.2021.04.070 34167646

[16] Chlebowski, R. T. Adriamycin (doxorubicin) cardiotoxicity: A review. *West J. Med.* **131**(5), 364–368 (1979). 394479 PMC1271861

[17] Siegel, D. A. et al. Pediatric cancer mortality and survival in the United States, 2001–2016. *Cancer.* **126**(19), 4379–4389 (2020). 32725630 10.1002/cncr.33080 PMC9539939

[18] Omland, T., Heck, S. L. & Gulati, G. The role of cardioprotection in cancer therapy cardiotoxicity. *JACC: Cardio Oncol.* **4**(1), 19–37 (2022). 10.1016/j.jaccao.2022.01.101 PMC9040117 35492815

[19] Kourek, C. et al. Cardioprotective Strategies from Cardiotoxicity in Cancer Patients: A Comprehensive Review. *J. Cardiovasc. Dev. Dis.* **9**(8), 259 (2022). 36005423 10.3390/jcdd9080259 PMC9409997

[20] Sławiński, G., Hawryszko, M., Liżewska-Springer, A., Nabiałek-Trojanowska, I. & Lewicka, E. Global longitudinal strain in cardio-oncology: A review. *Cancers (Basel).* **15**(3), 986 (2023). 36765941 10.3390/cancers15030986 PMC9913863

[21] Gonzalez-Manzanares, R. et al. Automated global longitudinal strain assessment in long-term survivors of childhood acute lymphoblastic leukemia. *Cancers (Basel).* **14**(6), 1513 (2022). 35326663 10.3390/cancers14061513 PMC8946759

[22] Niemelä, J. et al. Cardiac function after cardiotoxic treatments for childhood cancer-left ventricular longitudinal strain in screening. *Front. Cardiovasc. Med.* **8**, 715953 (2021). 34733890 10.3389/fcvm.2021.715953 PMC8558299

[23] Christiansen, J. R. et al. Utility of global longitudinal strain by echocardiography to detect left ventricular dysfunction in long-term adult survivors of childhood lymphoma and acute lymphoblastic leukemia. *Am. J. Cardiol.* **118**(3), 446–452 (2016). 27296561 10.1016/j.amjcard.2016.05.021

[24] Puckett, L. L. et al. Cardiotoxicity screening of long-term, breast cancer survivors-The CAROLE (Cardiac-Related Oncologic Late Effects) study. *Cancer Med.* **10**(15), 5051–5061 (2021). 34245128 10.1002/cam4.4037 PMC8335805

[25] Stokke, T. M. et al. Geometry as a confounder when assessing ventricular systolic function: comparison between ejection fraction and strain. *J. Am. Coll. Cardiol.* **70**(8), 942–954 (2017). 28818204 10.1016/j.jacc.2017.06.046

[26] Jefferies, J. L. et al. Cardiac remodeling after anthracycline and radiotherapy exposure in adult survivors of childhood cancer: A report from the St Jude lifetime cohort study. *Cancer.* **127**(24), 4646–4655 (2021). 34411296 10.1002/cncr.33860 PMC8664999

[27] Merkx, R. et al. Extensive cardiac function analyses using contemporary echocardiography in childhood cancer survivors: A Dccss Later study. *JACC CardioOncol.* **5**(4), 472–485 (2023). 37614574 10.1016/j.jaccao.2023.06.003 PMC10443197

